# Refractory Hailey–Hailey disease cleared with upadacitinib

**DOI:** 10.1016/j.jdcr.2023.09.011

**Published:** 2023-09-27

**Authors:** Lauren Murphy, Peter Ch’en, Eingun James Song

**Affiliations:** a University of Arizona College of Medicine, Tucson, Arizona; b Albert Einstein College of Medicine, Bronx, New York; c Frontier Dermatology, Mill Creek, Washington

**Keywords:** genodermatosis, Hailey–Hailey disease, JAK inhibitor, upadacitinib

## Introduction

Hailey–Hailey disease, also known as familial benign pemphigus, is a rare blistering genodermatosis. The genetic abnormality responsible for Hailey–Hailey disease is an autosomal dominant mutation in the ATP2Cl gene which codes a P-type Ca(2+)-transport ATPase.^1^ Impaired Ca(2+) signaling can lead to disrupted development of desmosomes of the epidermis, which results in acantholysis.^2^ Hailey–Hailey disease typically presents with painful, erythematous, and erosive lesions at sites of friction, including the inframammary regions, inguinal folds, and scrotum.^3^ Lesions consist of microscopically small flaccid vesicles on an erythematous base that eventually erode with crusting, scaling, and hypertrophic vegetative growths.^3^ Hailey–Hailey disease can significantly impair quality of life in patients affected by the disease because of pain, restriction of mobility, and cosmetic concerns. In addition to its negative effects on quality of life, Hailey–Hailey disease also increases infection risk in patients with the disease. Lesions in Hailey–Hailey disease are particularly vulnerable to colonization by *Staphylococcus aureus* and candida because of disruption of epidermal barrier function, which can further worsen acantholysis.^3^

There are a variety of treatments available for Hailey–Hailey disease, although treatment for many patients remains challenging and some patients are refractory to typical treatment modalities. Available treatment options include topical and systemic antimicrobial therapy, including tetracyclines, which are particularly effective.^3^ Additional treatment options include but are not limited to glucocorticoids, dermabrasion, carbon dioxide laser vaporization, naltrexone, and systemic retinoid.^3^

Upadacitinib is a second-generation JAK inhibitor, selective for the JAK1 enzyme. Upadacitinib has received US Food and Drug Administration approval for the treatment of psoriatic arthritis and atopic dermatitis.^4^ The utility of upadacitinib in the treatment of Hailey–Hailey disease has not been previously recorded in the literature. Upadacitinib has been successfully used to treat bullous pemphigoid, which is also an acantholytic disease.^5^ This case report highlights upadacitinib as a possible treatment option for patients with refractory Hailey–Hailey disease.

## Case presentation

**Fig 1:**
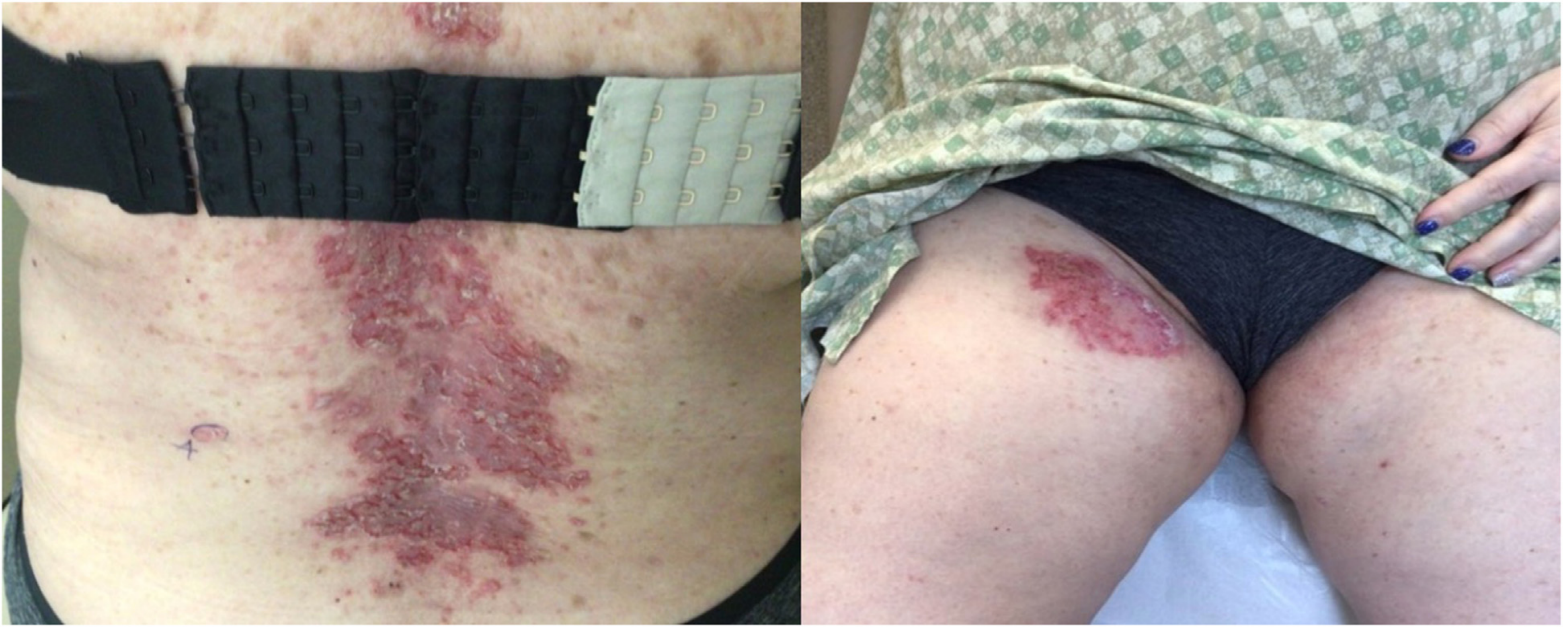
**A,B,** Before treatment with upadacitinib.

**Fig 2:**
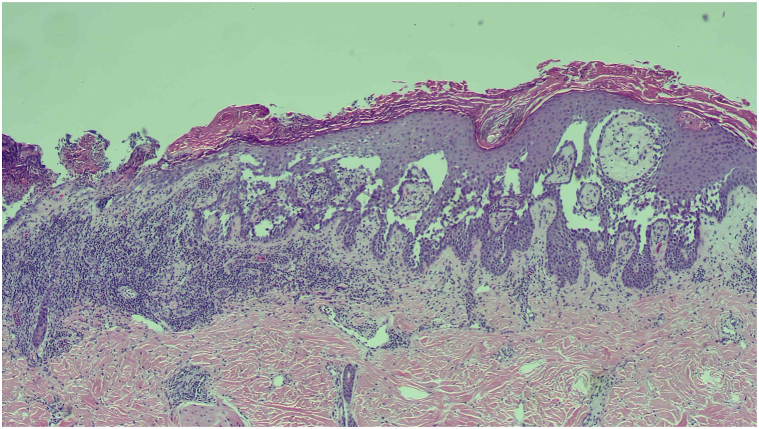
Shave biopsy consistent with Hailey–Hailey disease.

A 65-year-old White woman presented to our clinic for evaluation and treatment of widespread erythematous erosive plaques underneath her breasts, thighs, axilla, and lower portion of the back since early adulthood (Fig 1). Patient reported that her father also had a similar condition. Her medical history was notable for hypertension, hyperlipidemia, and depression. She was taking amlodipine 5 mg daily, atorvastatin 10 mg daily, and paroxetine 30 mg daily. A shave biopsy demonstrated dyskeratosis and elongated dermal papillae “villi” that extended into lacunae lined by a single layer of basal cells (Fig 2). This was compatible with a diagnosis of Hailey–Hailey disease. Patient was treated with triamcinolone, topical gentamicin, calcipotriene, minocycline, fluconazole, acitretin, dapsone, dupilumab, and low-dose naltrexone (titrated as high as 15 mg daily), which led to partial relief; however, she was never completely clear and continued to have severe flares several times a year. She was most recently treated with cyclosporine 300 mg daily, 0.1% gentamicin cream, and 0.1% triamcinolone cream, which were all stopped approximately 3 months before starting upadacitinib. She had the most improvement with intermittent courses of prednisone and cyclosporine but had to stop because of the risk of toxicity from long-term usage.

**Fig 3:**
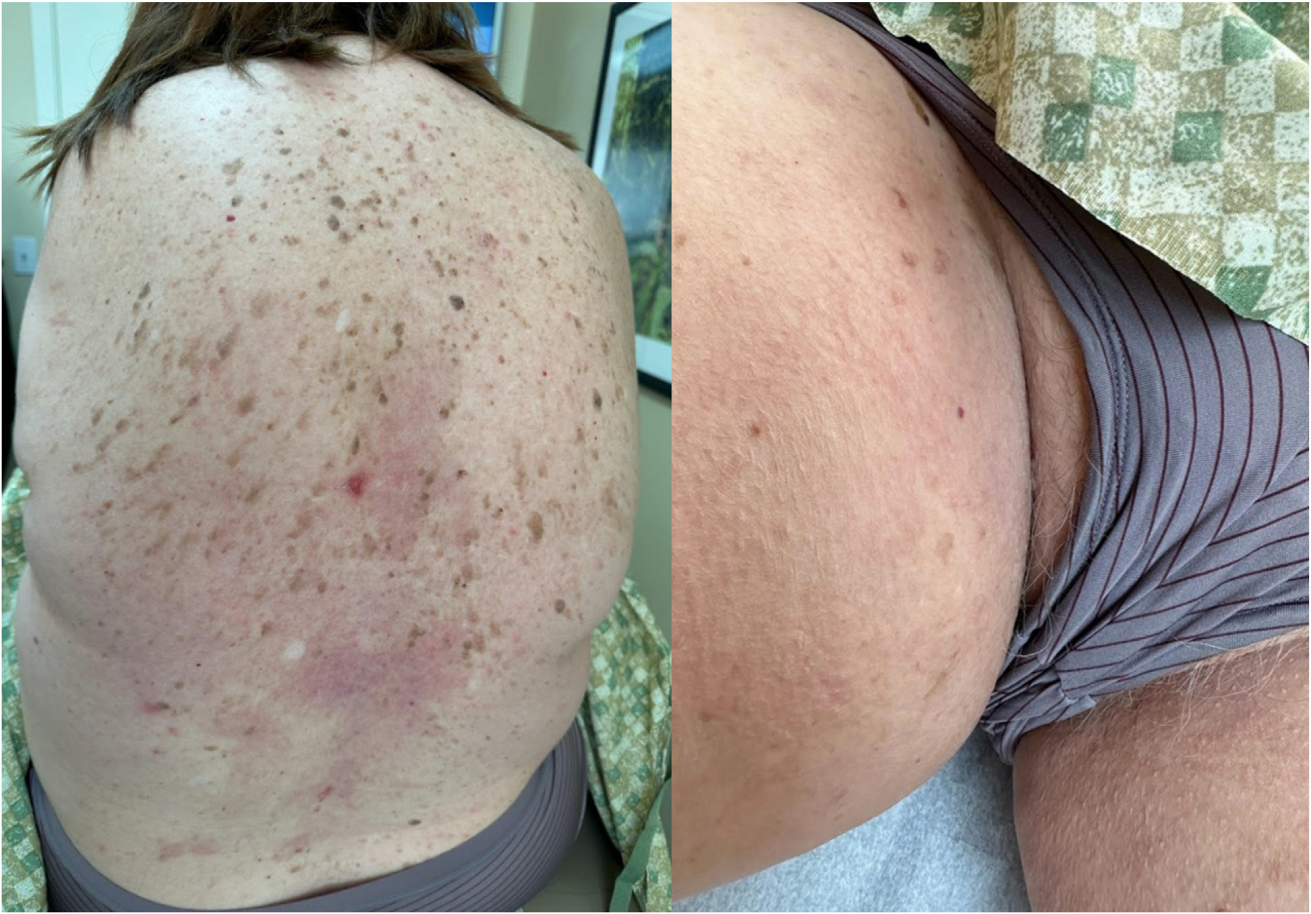
**A, B,** Response to upadactinib 15 mg daily after 12 weeks of treatment.

Having exhausted her treatment options, patient was agreeable to a trial of upadacitinib. The patient started treatment on 15 mg daily. The patient had all necessary laboratory tests completed before initiating treatment, including a complete blood cell count, complete metabolic panel, fasting lipid panel, hepatitis B surface antigen, hepatitis B surface antibody, hepatitis C virus, and QuantiFERON-TB Gold. Her baseline and repeated laboratory tests after 12 weeks of treatment were all within normal limits. She was also previously vaccinated with Shingrix. At her 4-week follow-up, all her active areas had healed with just some residual postinflammatory erythema (Fig 3). As of today’s writing, patient has remained clear for 16 weeks with no notable adverse events, including any significant laboratory changes.

## Discussion

Many cytokines and all interferons activate the JAKs and signal transducers and activators of transcriptions (STATs).^6^ Skin barrier defects associated with Hailey–Hailey disease may give rise to secondary Th2-mediated inflammation.^2^ The efficacy of treatments that target Th2-mediated pathways has been previously explored. Dupilumab has demonstrated efficacy in the treatment of Hailey–Hailey disease.^7^ The mechanism of dupilumab includes inhibition of both Th2-mediated interleukin (IL) 4 and IL-13 signaling.^8^ Previous research has explored the role that IL-4 and IL-13 may play in Ca(2+) mobilization in human keratinocytes.^9^ In one study, keratinocytes exposed to high levels of Ca(2+) resulted in a rapid mobilization of Ca(2+) into keratinocytes.^9^ In contrast, IL-4 and IL-13-treated cells showed a significant decrease in peak amplitude of Ca(2+) influx.^9^

Notably, Th2-mediated IL-4 and IL-13 are associated with the JAK1 and STAT6 signaling pathway.^6^ The role of upadacitinib in JAK1 inhibition could possibly explain the efficacy of upadacitinib observed in this case because inhibition of JAKs prevents inflammatory cytokines from utilizing the JAK/STAT pathway. As aforementioned, upadacitinib has already shown efficacy in treating bullous pemphigoid. Previous evidence also suggests that JAK inhibitors are efficacious in other inflammatory dermatoses, including atopic dermatitis, alopecia areata, psoriasis, and vitiligo.^10^ Although more research is needed to further elucidate the role of JAK inhibitors in the treatment of inflammatory dermatoses, the use of JAK inhibitors in these conditions is promising.

In conclusion, our case demonstrates the successful management of refractory Hailey–Hailey disease with upadacitinib, a novel JAK-1 inhibitor that has been approved for a variety of dermatologic indications. Although it is a singular case, it provides evidence for yet another potential use case for upadacitinib in the treatment of a historically difficult to treat dermatologic pathology.

## Conflicts of interest

AbbVie, Eli Lilly, Pfizer, Janssen, Amgen, BMS, Ortho-dermatologics, Sanofi & Regeneron, Arcutis, Dermavant, Incyte, SUN, UCB.
